# Structural Insights Into TDP-43 and Effects of Post-translational Modifications

**DOI:** 10.3389/fnmol.2019.00301

**Published:** 2019-12-17

**Authors:** Liberty François-Moutal, Samantha Perez-Miller, David D. Scott, Victor G. Miranda, Niloufar Mollasalehi, May Khanna

**Affiliations:** ^1^Department of Pharmacology, College of Medicine, University of Arizona, Tucson, AZ, United States; ^2^Center for Innovation in Brain Science, Tucson, AZ, United States; ^3^Department of Chemistry and Biochemistry, University of Arizona, Tucson, AZ, United States

**Keywords:** TDP-43 = TAR DNA–binding protein 43, structure, post-translational modification, subdomains, RRM domain

## Abstract

Transactive response DNA binding protein (TDP-43) is a key player in neurodegenerative diseases. In this review, we have gathered and presented structural information on the different regions of TDP-43 with high resolution structures available. A thorough understanding of TDP-43 structure, effect of modifications, aggregation and sites of localization is necessary as we develop therapeutic strategies targeting TDP-43 for neurodegenerative diseases. We discuss how different domains as well as post-translational modification may influence TDP-43 overall structure, aggregation and droplet formation. The primary aim of the review is to utilize structural insights as we develop an understanding of the deleterious behavior of TDP-43 and highlight locations of established and proposed post-translation modifications. TDP-43 structure and effect on localization is paralleled by many RNA-binding proteins and this review serves as an example of how structure may be modulated by numerous compounding elements.

## Introduction

Transactive response DNA binding protein (TDP-43), initially discovered in 1995 as a modulator of HIV-1 gene expression (Ou et al., 1995), is a highly conserved member of the heteronuclear ribonucleotide binding protein (hnRNP) family (Purice and Taylor, 2018). The TARDBP gene is located at chromosomal locus 1p36.22 and is comprised of six exons and while several TDP-43 isoforms have been identified, these have not been well-studied (Wang et al., 2004; Strong et al., 2007; D’Alton et al., 2015).

Since its discovery, TDP-43 has been shown to be involved in nearly all aspects of RNA metabolism (reviewed in Lagier-Tourenne et al., 2010; Ratti and Buratti, 2016) and is thought to be associated with more than 6000 RNA species. Of particular interest, TDP-43 has been shown to regulate its own mRNA (Ayala et al., 2011), the alternative splicing of hnRNPA1 mRNA (Deshaies et al., 2018), key cryptic exon splicing of *C9orf72* (Buratti et al., 2001) -the well-known hexanucleotide GGGGCC repeat expansion and the most frequent genetic cause of Amyotrophic Lateral Sclerosis (ALS) and Frontotemporal Lobar Degeneration (FTLD) (Balendra and Isaacs, 2018), Tau splicing resulting in accumulation of disease-associated isoform (Gu et al., 2017, 2018).

Although predominantly in the nucleus, TDP-43 is also present at low levels throughout the cell, including cytoplasm (Ayala et al., 2008) and mitochondria (Wang et al., 2016; Davis et al., 2018). Accumulation of insoluble, TDP-43-positive inclusions in the cytoplasm has emerged as a hallmark of ALS-FTLD. In these inclusions, TDP-43 is usually hyperphosphorylated, polyubiquitinated, and found as a mix of full-length and fragmented protein (Neumann et al., 2006, 2009; Nonaka et al., 2009b). Although it is generally accepted that TDP-43 localization to the cytoplasm is a mechanism of pathology, Moss et al. were not able to reproduce cellular pathologies by simply delocalizing TDP-43 to the cytoplasm (Wobst et al., 2017).

Although TDP-43 mutations are only associated with a small fraction of cases (Buratti, 2015), TDP-43-positive inclusions are found in the vast majority of postmortem neuronal tissue from confirmed ALS-FTLD patients (Arai et al., 2006; Neumann et al., 2009; Ling et al., 2013). Inclusions of similar histopathology are also found in other neurodegenerative disorders as well (Amador-Ortiz et al., 2007; Hasegawa et al., 2007; Leverenz et al., 2007; Nakashima-Yasuda et al., 2007; Geser et al., 2008). Additionally, both loss and excess of TDP-43 are toxic, confirming that this is an essential protein and suggesting tight regulation of expression and localization (Kraemer et al., 2010; Wu et al., 2010; Ayala et al., 2011; Budini and Buratti, 2011; Polymenidou et al., 2011; Wegorzewska and Baloh, 2011). However, despite intense research, there is no clear consensus about what causes TDP-43 mislocalization and aggregation, nor how it contributes to neuronal toxicity. For potential pathways involved in the aberrant behavior of TDP-43 we refer the reader to several recent reviews (Janssens and Van Broeckhoven, 2013; Prasad et al., 2019).

Like other RNA binding proteins (RBPs), TDP-43 has a beads-on-a string architecture consisting of multiple, independent functional domains and many sites of post-translational modification. Despite its relatively small size (43 kDa), structural determination of the full-length protein has been a challenge, likely due to the large portion of the protein that is intrinsically disordered. Estimates of intrinsically disordered regions (IDR) range from 15–30% of the total protein and 36–66% of the C-terminal domain (Mészáros et al., 2018). Nevertheless, 3-dimensional structures of the N-terminal domain, the RNA-binding domains, and segments of the C-terminal domain have become available in recent years. The primary aim of this review is to consolidate the insights that these structures bring to our developing understanding of the functions and deleterious behavior of TDP-43 and to highlight the location of both established and proposed post-translational modifications.

### Structure Overview

**FIGURE 1:**
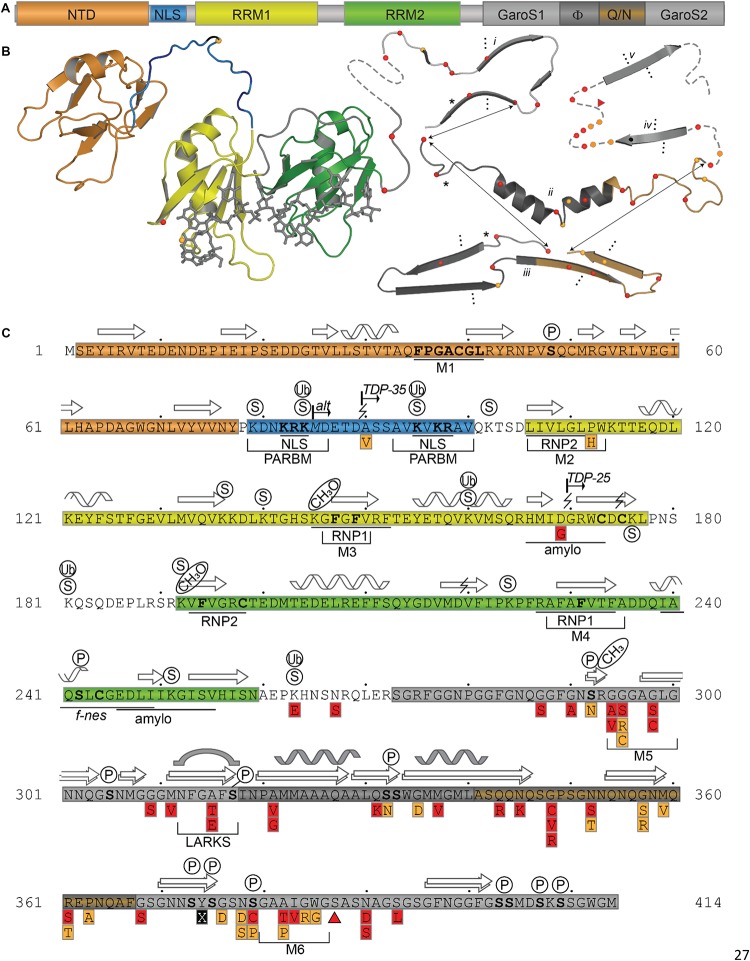
TDP-43 Structure and Sequence Features. **(A)** Domain map showing relative sizes of the N-terminal domain (NTD, *orange*), linker containing nuclear localization signal (NLS, *blue*), RNA-recognition motif 1 (RRM1, *yellow*), RNA-recognition motif 2 (RRM2, *green*), and the C-terminal subdomains identified by Mompeán et al. (2016a): Gly-aromatic-Ser-rich regions (GaroS, *light gray*), hydrophobic region (Φ, dark gray), glutamine-arginine-rich region (Q/N, *orange gray*). **(B)** Representative structures with variant sites shown as spheres and colored as in panels A and C. NTD (5mrg), RRM1-RRM2 with RNA as gray sticks (4bs2), CTD fragments *i* [6n3c (Cao et al., 2019)], *ii* [2n3x (Jiang et al., 2016)], *iii* [6n3a (Guenther et al., 2018a)], *iv* [5wia (Guenther et al., 2018a)], *v* [5wiq (Guenther et al., 2018a)]. Asterisk marks LARKS/Omega Loop. Arrows indicate residues that correspond among three shown polymorphs. Ellipses indicate tendency to form fibrils (*i, iii*) or steric zipper structures (*iv, v*). **(C)** Annotated primary sequence. Post-translational modifications shown in circles above sequence (P, phosphorylation site PhosphoSitePlus (Hornbeck et al., 2015)]; S, SUMOylation sites identified by high-throughput studies (Hendriks et al., 2015, 2017); CH3, monomethylation site (Guo et al., 2014). Alternative start site at M85 [alt (Nishimoto et al., 2010)]. Caspase digestion sites [lightning bolts (Zhang et al., 2007; Rohn, 2008; Li et al., 2015; Chiang et al., 2016)]. Proposed mitochondrial targeting sequences (M1, M3, and M5) (Wang et al., 2016). Bipartite nuclear localization signal (NLS) (Winton et al., 2008) overlapping with the Poly-ADP-ribose (PAR) binding motif (PARBM) (McGurk et al., 2018). Conserved ribonucleotide interacting motifs (RNP1, RNP2) (Buratti et al., 2001). Amyloidogenic regions (amylo) (Shodai et al., 2012, 2013). Non-functional (former) nuclear export signal [f-nes (Archbold et al., 2018; Ederle et al., 2018; Pinarbasi et al., 2018)]. Sequence variants compiled using the ClinVar database (April 2019) (Landrum et al., 2016) and a review by Buratti et al. (Buratti, 2015), shown in red boxes if linked to disease, orange if clinical significance is not clear. Site of insertion/deletion [triangle (Solski et al., 2012)] and premature stop (X) also shown. Dots above sequence mark every 10 residues.

TDP-43 is composed of a well folded N-terminal domain (NTD), two highly conserved RNA recognition motifs (RRM1 and RRM2), and a glycine-rich C-terminal domain (Figure 1).

#### The N-Terminal Domain and Nuclear Localization Signal

##### Organization/oligomerization of the N-terminal domain (NTD, amino acids 1–77)

The TDP-43 N-terminal domain has been shown to form dimers and higher-order oligomers both *in vitro* and in the cell (Shiina et al., 2010; Chang et al., 2012; Wang et al., 2013; Zhang et al., 2013; Afroz et al., 2017; Jiang et al., 2017; Mompeán et al., 2017; Sun et al., 2018; Wang A. et al., 2018) with a single dissociation constant of approximately 70 μM, indicating absence of cooperativity in binding of subunits (Wang A. et al., 2018). Unlike pathological aggregates, which are hyperphosphorylated and ubiquitinated (Arai et al., 2006; Hasegawa et al., 2008), reversible formation of TDP-43 polymers through the NTD has been shown to be required for splicing activity (Afroz et al., 2017; Jiang et al., 2017; Mompeán et al., 2017; Wang A. et al., 2018) and to contribute to phase separation *via* liquid-droplet formation (Afroz et al., 2017; Wang A. et al., 2018), thought to contribute to formation of cytoplasmic stress granules (SGs) (Molliex et al., 2015). The NTD is also the site of one of three predicted mitochondrial targeting sequences (Wang et al., 2016), conserved phosphosite Ser 48 (Rigbolt et al., 2011; Wang A. et al., 2018), as well as potential, albeit weak, nucleotide binding (Chang et al., 2012; Qin et al., 2014; Mompeán et al., 2016c; Wang L. et al., 2018). Thus far, five 3-dimensional structures of the NTD have been published, including three monomeric (Mompeán et al., 2016c, 2017; Jiang et al., 2017) and two dimeric structures (Afroz et al., 2017; Wang A. et al., 2018). Structural alignment shows that the overall folds are highly similar, with backbone root-mean-square deviations (RMSD) of 0.5–2.5 Å for individual subunits (Table 1).

**TABLE 1:** TDP-43 domain structures.

**PDB code**	**Technique (resolution)**	**Observed amino acids**	**Notes**	**Observed oligomeric state**	**Backbone RMSD**	**References**
**N-terminal domain**						
2n4p	NMR	1–77	6-His tag	Monomer	0.46	Mompeán et al., 2016c
5mdi	X-ray (2.1 Å)	2–79	6-His tag, C39/C50-dimethylarsinoyl	Dimer	1.50 (A) 1.63 (B)	Afroz et al., 2017
5mrg	NMR	1–102	6-His tag	Monomer	–	Mompeán et al., 2017
5 × 4f	NMR	1–77	C39/C50S, GB1 tag	Monomer	2.43	Jiang et al., 2017
6b1g	NMR	1–80	Y4R (A), S48E (B)	Dimer	1.78 (A) 1.45 (B)	Wang A. et al., 2018
**RRM domains**						
4bs2	NMR	102–269 (RRM1-RRM2)	With UG-rich RNA 5′-R(^∗^GP^∗^UP^∗^GP^∗^UP^∗^GP^∗^AP^∗^AP^∗^ UP^∗^GP^∗^AP^∗^AP^∗^UP)-3′	Monomer		Lukavsky et al., 2013
2cqg	NMR	96–185 (RRM1)		Monomer	1.37a	–
4iuf	X-ray (2.75 Å)	103–179 (RRM1)	With 5′-D(^∗^GP^∗^TP^∗^TP^∗^GP^∗^(XUA)P^∗^GP^∗^ CP^∗^GP^∗^T)-3′	Monomer	1.02a	Kuo et al., 2014
4y00	X-ray (3.00 Å)	103–168(RRM1)	D169G, with (5′-D(P^∗^TP^∗^TP^∗^GP^∗^AP^∗^GP^∗^CP^∗^ GP^∗^T)-3′)	Monomer	0.97a	Chiang et al., 2016
4y0f	X-ray (2.65 Å)	103–180 (RRM1)	With (5′-D(^∗^GP^∗^TP^∗^TP^∗^GP^∗^AP^∗^GP^∗^CP^∗^ GP^∗^TP^∗^T)-3′)	Monomer	1.00a	Chiang et al., 2016
1wf0	NMR	193–267 (RRM2)	E200G	Monomer	1.13b	–
3d2w	X-ray (1.65 Å)	192–261 (RRM2 mouse)	With (5′-D(^∗^DGP^∗^DTP^∗^DTP^∗^DGP^∗^DAP^∗^ DGP^∗^DCP^∗^DGP^∗^DTP^∗^DT)-3’)	Homo 2-mer	0.84b	Kuo et al., 2009
**C-terminal domain**						
2n2c	NMR	307–348		Monomer		–
2n3x	NMR	311–360		Monomer		Jiang et al., 2016
2n4g	NMR	311–360	G335D	Monomer		Jiang et al., 2016
2n4h	NMR	311–360	Q343R	Monomer		Jiang et al., 2016
5whn	X-ray (1.1 Å)	312–317		Homo 15-mer		Guenther et al., 2018a
5whp	X-ray (1.0 Å)	312–317	A315T	Homo 10-mer		Guenther et al., 2018a
5wia	X-ray (1.0 Å)	370–375		Homo 10-mer		Guenther et al., 2018a
5wiq	X-ray (1.25 Å)	396–402		Homo 10-mer		Guenther et al., 2018a
5wkb	Electron crystallography (1.0 Å)	312–317	A315E	Homo 10-mer		Guenther et al., 2018a
5wkd	X-ray (1.8 Å)	300–306		Homo 10-mer		Guenther et al., 2018a
6cb9	X-ray (1.1 Å)	328–333		Homo 10-mer		Guenther et al., 2018a
6cew	X-ray (1.2 Å)	321–326		Homo 12-mer		Guenther et al., 2018a
6cf4	Electron crystallography (0.75 Å)	312–317	Phosphorylated A315T	Homo 10-mer		Guenther et al., 2018a
6cfh	Electron crystallography (1.5 Å)	333–343		Homo 20-mer		Guenther et al., 2018a
6n3a	Electron microscopy (3.3 Å)	311–360		Homo 10-mer		Cao et al., 2019
6n3b	Electron microscopy (3.8 Å)	312–352		Homo 10-mer		Cao et al., 2019
6n3c	Electron microscopy (3.3 Å)	288–314	A315E	Homo 20-mer		Cao et al., 2019
6n37	Electron microscopy (3.8 Å)	312–347		Homo 10-mer		Cao et al., 2019
**RRM2 amyloidogenic core**						
5w50	X-ray (1.4 Å)	248–253		Homo 12-mer		Guenther et al., 2018b
5w52	Electron crystallography (1.4 Å)	247–257		Homo 10-mer		Guenther et al., 2018b
5w7v	Electron microscopy (3.8 Å)	247–257		Triple- helical fibril		Guenther et al., 2018b

*^∗^Structural alignment of all backbone atoms reported as the root-mean-square deviation (RMSD) in Å. NTD alignment to 5mrg core residues 1–77. RRM alignment to 4bs2 RRM1 (102–179)^*a*^ or 4bs2 RRM2 (190–262)^*b*^. In the case of multiple copies in the asymmetric unit, the average RMSD is reported.*

**FIGURE 2:**
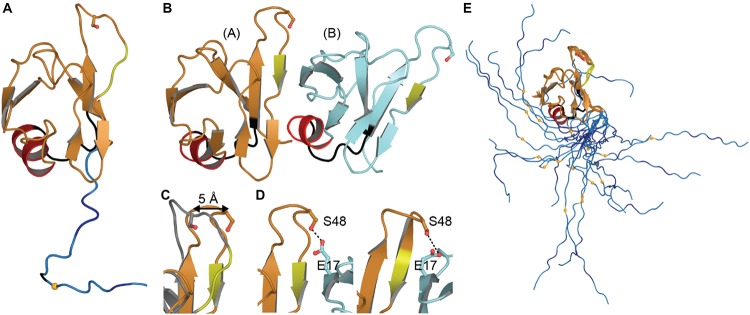
NTD Structures. **(A)** Monomeric structure shown in ribbon representation with location of mitochondrial targeting sequence 1 in black, residues involved in loop-sheet transition during dimer formation in yellow, Ser 48 in sticks representation, residues 97–102 in blue with A90 as a small orange sphere. **(B)** Dimeric X-ray structure with features as in A. **(C)** Overlay of monomer (*gray*) and subunit A of dimeric structure (*orange*) showing shift of S48 Cα and loop to β-strand transition (*yellow*). **(D)** Inter-subunit interactions of Ser 48 in dimeric X-ray (*left*) and NMR (*right*) structures. Dashes show contacts within hydrogen bonding distance. **(E)** NMR ensemble of NTD monomer showing varied position of the NLS. Coloring as in panel **(A)**. Monomeric NMR structure PDB ID 5mrg (Mompeán et al., 2017), dimeric X-ray structure PDB ID 5mdi (Afroz et al., 2017) and dimeric NMR structure PDB ID 6b1g (Wang A. et al., 2018).

The NTD monomer consists of six β-strands and a single α-helix arranged in a ubiquitin-like β-grasp fold, similar to the DIX domain of Axin 1 (Mompeán et al., 2016c; Figure 2A). The DIX domain is known to facilitate both homo- and hetero-oligomerization (Kishida et al., 2015). Consistent with this, the dimeric NTD structures revealed a head-to-tail configuration of subunits (Afroz et al., 2017; Wang A. et al., 2018; Figure 2B). As noted by Wang A. et al. (2018) these two structures are very similar overall, differing by a moderate rotation and shift between subunits, likely due to differences in experimental conditions. In both cases, the interface is formed mainly by charged and polar residues (Afroz et al., 2017; Wang A. et al., 2018). Comparison with the monomeric structures shows that upon dimerization, a surface-accessible and mobile loop becomes more structured as it participates in the interface (Figures 2A,B). Phosphosite Ser 48 is also shifted toward the interface where it is involved in hydrogen bonding interactions (Figures 2C,D). It has been shown that mimicking phosphorylation at this site disrupts self-association of the NTD and affects splicing function (Wang A. et al., 2018). Additionally, one of three mitochondrial targeting sequences (F35 – L41) occurs in the loop following the α-helix (Figures 2A,B), discussed in more detail below (see section “Mitochondrial Targeting”). Finally, crystal-packing symmetry in the X-ray structure suggests that the NTD alone can form a superhelical structure (Afroz et al., 2017), but the relevance of this supra-assembly for higher-order oligomerization of the full-length protein is unclear.

##### The TDP-43 linker and nuclear localization signal (NLS, amino acids 78–100)

The canonical, positively charged, bi-dentate nuclear localization signal (NLS, Figure 1) occurs in the linker between the NTD and the RNA-binding domains. The NLS is recognized by Importin-α for active transport of TDP-43 into the nucleus (Winton et al., 2008; Nishimura et al., 2010; Archbold et al., 2018; Pinarbasi et al., 2018). However, the NLS can be disrupted by caspase cleavage at Asp 89 (Suzuki et al., 2011) or by initiation at the alternative start site, Met 85 (Nishimoto et al., 2010; Xiao et al., 2015) resulting in accumulation of TDP-43 in the cytoplasm, an event generally agreed to result in formation of insoluble aggregates (Winton et al., 2008; Shenouda et al., 2018). In one study, the SNP A90V was shown to result in mild disruption of the NLS and low-level mislocalization (Winton et al., 2008). However, this SNP was also identified in one healthy control in the same study and two additional healthy persons in another study (Sreedharan et al., 2008), leaving the clinical significance of this substitution in doubt.

Only one structure to date contains the entire linker and this structure shows that the NLS is quite mobile (Figure 2E). The flexibility of this linker leads us to speculate that the connection between the NTD and RNA-binding domains is dynamic, allowing the arrangement of these domains to change with NTD-self association, RNA binding, post-translational modifications, and/or interactions with other proteins.

##### Nucleic acid binding by the NTD and linker

Several studies have hinted at the ability of TDP-43 NTD to interact with nucleic acids (Chang et al., 2012; Qin et al., 2014; Mompeán et al., 2016c). While Chang et al. (2012) did not find a significant binding for TDP-43-NTD_1__–__105_ to single stranded DNA (TG)_6_, the presence of TDP-43-NTD_1__–__105_ did increase by three-fold the affinity of TDP-43 for this nucleic acid sequence compared to RRMs only. An independent study proposed, using HSQC-NMR, the ability of the folded form to bind ssDNA (TG)_6_, RNA (UG)_6_ but not ss(TT)_6_, suggesting a specificity component in the binding (Qin et al., 2014). While Qin et al. (2014) did not observe a binding event implicating the NLS (81–102), a third study showed a binding of this positively charged region to both ss(TG)_6_ and ss(TT)_6_ and report a Kd < 150 μM (Mompeán et al., 2016c). Even though the nucleotide binding interface of the NTD remains to be determined, it seems clear that the TDP-43 NTD and NLS serve as a structural support for nucleic acid binding and might contribute to specificity toward certain nucleic acid sequences.

#### The RNA-Recognition Motifs (RRM1-RRM2)

##### RRM-nucleic acid binding

The RNA-recognition motifs of TDP-43 span amino acids 106–177 (RRM1) and 192–259 (RRM2), each of which contain two highly conserved short sequence motifs known as RNP-1 (octameric sequence: KGFGFVRF in RRM1 and RAFAFVTF in RRM2) and RNP-2 (hexameric sequence: LIVLGL in RRM1 and VFVGRC in RRM2) (Figure 1) required for nucleic acid binding. To date, there are four structures of RRM1, two structures of RRM2 and one of the tandem RRM1-RRM2. These structures are highly similar, with RMSD ranging from 0.84 to 1.37 Å (Table 1).

**FIGURE 3:**
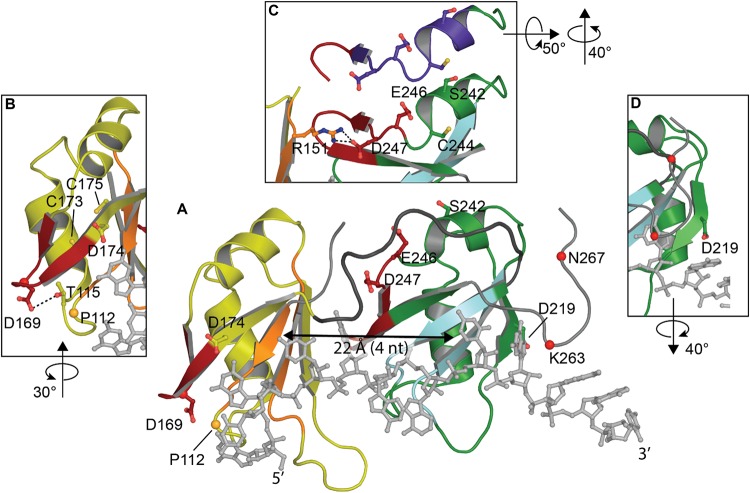
Structure of TDP-43 RRM domains. **(A)** Overview showing RRM1 in yellow with RNP-1 and RNP-2 in orange, flexible linker in dark gray, RRM2 in green with RNP-1 and RNP-2 in cyan and UG-rich RNA in light gray. **(B)** Rotated view of amyloidogenic core residues 166–173. **(C)** Overlap between amyloidogenic core residues 246–255 (red in view **A)** with offset showing former NES in purple. **(D)** Rotated view of cleavage site D219 [PDB ID 4bs2 (Lukavsky et al., 2013)].

Both RRMs fold into a 5-stranded beta sheet stacked against two alpha helices with the conserved RNA-binding aromatic and hydrophobic residues located in the β-sheets, shown to form stacking interactions with the bases and the sugar rings of single-stranded RNA or DNA. These tandem RRM domains bind nucleic acid in the 5′ to 3′ direction, opposite to typical tandem RRMs (Lukavsky et al., 2013; Kuo et al., 2014; Figure 3A). Interestingly, compared to typical 4-stranded RRMs, the TDP-43 RRM1 and RRM2 both contain a supplementary β-strand thought to be necessary for structure stability (Shodai et al., 2013).

Studies have consistently shown preferential binding of TDP-43 to (TG)/(UG) sequences (Buratti et al., 2001; Kuo et al., 2009; Sephton et al., 2011; Colombrita et al., 2012; Bhardwaj et al., 2013; Lukavsky et al., 2013; Furukawa et al., 2016). RRM1 is known to be sufficient and essential for proper nucleic acid binding as its affinity is in the low nanomolar range for the canonical UG-rich sequence, while RRM2 is not (Kuo et al., 2009; Furukawa et al., 2016). RRM1 was shown to be able to bind other motifs, consistent with (Kuo et al., 2014), A3(GG)4A3, *via* a distinct binding site implicating Arg151 (Sephton et al., 2011). R151A did not impact sequence recognition of the RRM1-RRM2 tandem construct while substitution of E246 or D247 (Lukavsky et al., 2013) were able to decrease the specificity for RNA sequences. The authors concluded that E246 and especially D247 were crucial in the structure of RRM2 and the nucleic acid binding. Indeed, D247 is stabilizing the RRM1-RRM2 orientation when RNA is bound to the protein by making a salt bridge with Arg151 of RRM1 (Lukavsky et al., 2013; Figure 3C).

The recognition of nucleic acid sequences is achieved by an interaction between the two RRMs, which is typical of proteins that contain several RRMs, and RRM2 is being considered as a switch to regulate the sequence-specificity in the substrate binding of TDP-43 by hiding Arg151 (Sephton et al., 2011). Cooperation between RRM1 and RRM2 also explains why TDP-43 affinity for nucleic acids increases with the length. Kuo et al. reported a large affinity increase between 4 and 6 nucleotides long, which approximately corresponds to the distance between both nucleic acid binding sites (∼22 Å) (Shodai et al., 2013; Figure 3A). Additionally, the RRMs are connected by a highly flexible loop (178–191), thought to confer adaptability to different nucleic acid partners by allowing different orientations of the RRM domains (Auweter et al., 2006; Furukawa et al., 2016).

Although it is believed that TDP-43 binds thousands of different transcripts, there are only co-structures with UG or TG repeat RNA and DNA. Given that RRM1 was suggested to bind non-UG/TG rich sequences *via* a site distinct from RNP sequences, structural characterization of TDP-43 with non-canonical motifs might yield interesting novel data on TDP-43 binding to nucleic acid.

##### Amyloidogenic cores

Although RRM1-2 are structurally well-folded and mostly known for interaction with nucleic acids, several studies have now suggested the presence of amyloidogenic cores and the ability of these domains to misfold and participate in either nucleation or propagation of TDP-43 aggregation in ALS (Shodai et al., 2012, 2013). Two regions of interest have been identified in ALS patients but not in well-folded non-pathological TDP-43: residues 166–173 in RRM1 (Shodai et al., 2013) and 246–255 in RRM2 (Shodai et al., 2012).

Residues 166–173 in RRM1 occur in a reasonably accessible loop (50% accessibility) (Figure 3B). Intriguingly, one of the two disease linked mutation in the RRM domain is D169G. While this mutation had no effect on TDP-43 RNA binding, the mutation stabilized TDP-43 and induced a loss of hydrogen bond with T115 resulting in a local conformational change (Chiang et al., 2016). D169G seemed to have no impact on aggregate formation but rather increased caspase cleavage (Shodai et al., 2013; Chiang et al., 2016).

Residues 246–255 in RRM2 lie between RRM1 and RRM2 and are not exposed in the folded state (27% accessibility) (Figure 3C). *In vitro* experiments have shown the ability of peptides encompassing residues 246–255 to participate in the formation fibrillar structures (Saini and Chauhan, 2011). The minimal sequence EDLIIKGISV is necessary and deletion of the first as well as the second residues resulted in reduced aggregation (Saini and Chauhan, 2011). Moreover, substitution of E246 or D247 to glycine residues in the RRM2 protein induced the formation of fibrils (Shodai et al., 2012). A recent structural study showed the ability of 247-DLIIKGISVHI-257 peptide to present amyloid polymorphism characterized by different backbone conformations as well as seven distinct steric zipper arrangements, a common structural feature of amyloid proteins (Guenther et al., 2018b; Table 1).

Interestingly, those two regions, 166–173 and 246–255, are the target of several pathological modifications- caspase cleavage, oxidation, ubiquitination. Those post-translational modifications will be discussed later in the manuscript (see section “Post-translational Modifications”).

##### Former NES

Until 2018, TDP-43 was thought to have a Nuclear Export Signal in RRM2: 239-IAQSLCGEDLI-249, that was predicted to be a substrate of the nuclear export factor exportin-1 (XPO1/CRM1) (Ayala et al., 2008; Winton et al., 2008). The lack of experimental validation, the poor fit of this sequence to the XPO1/CRM1 consensus sequence (Φ1-X2,3-Φ2-X2,3-Φ3-X-Φ4, where Φn represents Leu, Val, Ile, Phe, or Met and X can be any amino acid), the fact that most validated NES are found in unstructured regions, contrary to the location within the well-folded RRM2, and the limited surface accessibility of some residues in TDP-43 NES sequence, led three independent groups to further investigate TDP-43 export to cytoplasm. They all found that (*i*) inhibition of XPO1 either by siRNA or inhibitors (leptomycin B - LMB-, as well as selective inhibitors of nuclear export -SINE) or overexpression of XPO1 had no effect on TDP-43 localization to cytoplasm, (*ii*) TDP-43 NES fused to GFP or YFP did not induce a specific localization to cytoplasm of the constructs (Archbold et al., 2018; Ederle et al., 2018; Pinarbasi et al., 2018). Moreover, Pinarbasi et al. (2018) showed weak affinity of TDP-43 “NES” for XPO1, in the micromolar range. While Archbold et al. (2018) suggested, with limited effect, a redundant mechanism of active nuclear export, implicating XPO7 (Exportin 7) and NXF1 (Nuclear RNA export factor 1), both Ederle et al. (2018) and Pinarbasi et al. (2018) showed a passive diffusion mechanism, slowed down by size and inhibited by newly synthesized RNA binding.

Interestingly, mutations in TDP-43 NTD, L27A and L28A, both leading to monomeric TDP-43, decreased and abolished TDP-43 splicing function, respectively, and induced a partial or complete cytoplasm localization (Mompeán et al., 2017). Foglieni et al. (2017) also showed, using split-GFP technology which allows detection of weak and/or transient species, that dimeric TDP-43 was only nuclear. Since passive retention in the nucleus was recently suggested as the preferred way to retain proteins in the nucleus (Wühr et al., 2015), it is likely that dimeric species of TDP-43 bound to RNA and other RNA related machineries will be nuclear and the free monomeric protein will be able to diffuse out to the cytoplasm. This may explain how TDP-43 shuttles between nucleus and cytoplasm in a transcription-dependent manner (Ayala et al., 2008).

In light of this, results showing that the TDP-43-”ΔNES” (I239A/L243A,L248A/I249A/I250A) did not induce TDP-43-related toxicity in Drosophila and cell lines (Winton et al., 2008; Ritson et al., 2010) remain puzzling. Pinarbasi et al. (2018) suggested that contrary to early hypothesis stipulating that TDP-43-ΔNES inhibited TDP-43 cytoplasmic distribution, the neuroprotective effect of TDP-43-ΔNES arises from the disruption of TDP-43 splicing function. The authors compared TDP-43-ΔNES to the F147/149 L mutation, also able to disrupt splicing function and to alleviate overexpressed TDP-43 toxicity in *D. melanogaster*. This might seem surprising at first, since, contrary to F147 and F149, I239, L243, L248, I249, and I250 are not localized at the RNA interface (Figure 3C). But, those residues are localized at the interface between RRM1 and RRM2 and any modification of those contacts seems to lead to loss of RNA binding, similar to D247 mutations. However, E246 and D247 mutations resulted in a misfolded TDP-43 protein, reduced TDP-43 solubility and induced protein aggregation (Shodai et al., 2012).

**FIGURE 4:**
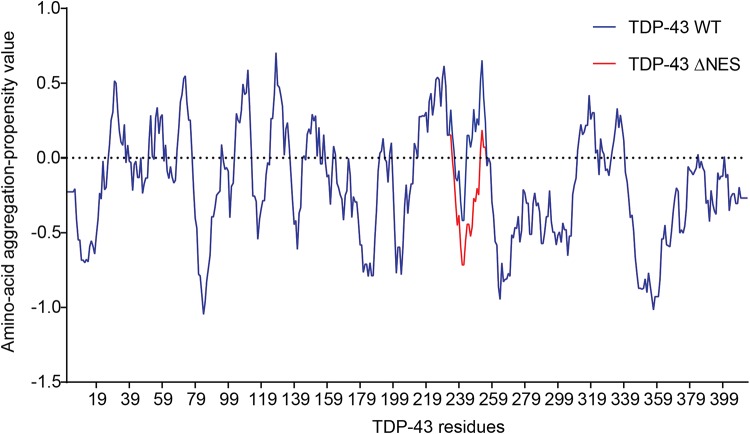
Prediction of amyloidogenicity of TDP-43 and TDP-43-”ΔNES.” Those mutations reduce the aggregation prone characteristic of the 244–255 region.

Since part of the “NES” overlaps with the amyloidogenic core 2 (residues 246–255, Figure 3C), we hypothesized those mutations would modify the amyloidogenicity of this sequence. We used Aggrescan, an online software that predicts amyloidogenic cores and was previously used on TDP-43 (Garnier et al., 2017). The comparison of TDP-43-WT versus TDP-43-”ΔNES” shows a suppression of the known amyloidogenic core at residues 244–255 (Figure 4), exposed in ALS patients (Saini and Chauhan, 2011; Shodai et al., 2012). Nevertheless, without thorough structural analysis of the effects of those mutations on TDP-43, no clear conclusions can be drawn.

#### The Aggregation-Prone C-Terminal Domain (CTD, Amino Acids 260–414)

TDP-43 CTD is the site of about 50 disease-linked mutations (Mackenzie and Rademakers, 2008) as well as most of the phosphorylation sites (Figure 1), and has thus been examined extensively, but due to its disordered nature, all structural studies of the CTD have been of fragments. The TDP-43 CTD has been shown to be required for TDP-43 splicing activity (Ayala et al., 2005; Freibaum et al., 2010; Conicella et al., 2016), including autoregulation (Ayala et al., 2011), and is the site of interaction with several protein partners such as UBQLN2 (Cassel and Reitz, 2013), FMRP (Majumder et al., 2016) and hnRNP (Buratti et al., 2005; D’Ambrogio et al., 2009).

##### Organization of the CTD

The CTD is able to form secondary structural elements that have been observed by crystallography and electron microscopy (Table 1) and distinct subdomains have been established: two Gly-aromatic-Ser-rich (GaroS) regions, and an amyloidogenic core divided into a hydrophobic region, and Q/N-rich region (Mompeán et al., 2016a; Figure 1).

The GaroS regions (residues 273–317 and 368–414) are similar to regions in FUS (Murray et al., 2017) proposed to interact within RNA granules (Mompeán et al., 2016a) and contributing to the formation of hydrogels (Kato et al., 2012).

**FIGURE 5:**
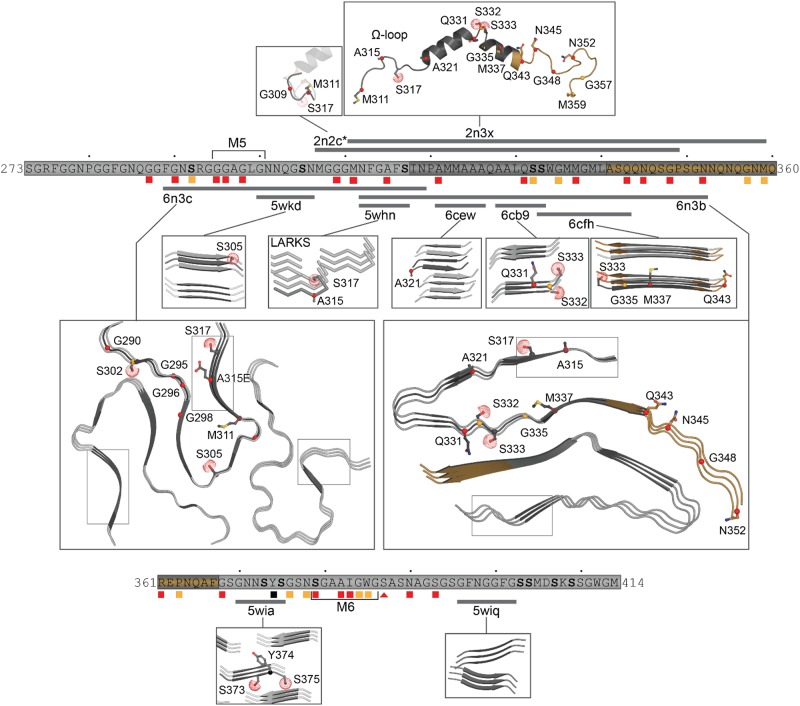
Structures of the C-terminal domain. Structures shown with mutation sites as sticks and phosphorylation sites with dots at the SG atom. Simplified primary sequence annotations as in Figure 1. Structure 2n2c (Lim et al., 2016) is highly similar to that of 2n3x (Jiang et al., 2016), thus only a portion is shown here, highlighting the non-overlapping residues M307-G310. Representatives of the short segments that form steric zippers or LARKS (5wkd, 5whn, 6cew, 6cb9, 6cfh, 5wia, 5wiq) (Guenther et al., 2018a) are shown in darker gray with neighboring strands as determined by symmetry in lighter gray. The R-shaped polymorph (6n3c) (Cao et al., 2019) with chain A in darker gray and only three filaments and immediate adjacent layers shown for clarity. A representative dagger-shaped fibril-forming polymorph (6n3a) (Cao et al., 2019) is shown with chain B in darker gray with two filaments and immediate adjacent layers. Note different shapes of LARKS segments within 6n3c and 6n3b (thin boxes). For clarity, mutation or phosphorylation sites shown only on the primary strand in all images.

The hydrophobic region (residues 318–340) can adopt a helical structure or Thioflavin T-positive filaments consistent with cross-β architecture (Jiang et al., 2013, 2016; Mompeán et al., 2014; Lim et al., 2016). The first structure determined in a lipid-like environment showed an omega-loop-helix structure (Ω-loop-helix, residues 320–343) (Lim et al., 2016). This was confirmed with a slightly longer construct determined under non-lipid conditions that showed a helix-turn-helix (Figure 5) that was disrupted by G335D and Q343R mutations (Jiang et al., 2016).

Likewise, Q/N rich regions (residues 341–369) were observed to form amyloid and amyloid-like aggregates (Mompeán et al., 2014). More recent structural studies identified six segments that form classic steric zippers and contribute to pathogenic aggregation (Guenther et al., 2018a; Figure 5), confirming the helix-to-sheet transition of the hydrophobic region and identifying additional aggregation-prone segments. This study also showed that the Omega-loop region, required for neurotoxicity (Zhu et al., 2014) can form Low complexity Aromatic-Rick Kinked Structures (LARKS) which can be affected by mutation and phosphorylation (Guenther et al., 2018a, b). These segments stack into kinked beta sheets, forming reversible (Velcro-like) associations thought to play a role in protein interactions and were hypothesized to play a role in reversible association of the CTD as well as pathogenic aggregation by bringing adjacent amyloid-forming segments together (Guenther et al., 2018a). Finally, multiple lines of evidence strongly suggest that the Q/N region also forms extended β-hairpin structures (Mompeán et al., 2014, 2015, 2016a; Cao et al., 2019). This was confirmed by very recent work describing the structures of two significantly longer regions using cryo-EM, both of which form irreversible fibrils (Cao et al., 2019). The first segment (residues 286–331), contains an ALS linked mutation (A315E) and folds into an R-shaped motif (Figure 5). The second segment (residues 311–360) exhibited three polymorphs with different inter-fibril contacts, but all sharing a common dagger-shaped motif at the core (Figure 5). Interestingly, structural alignment of the overlapping regions resulted in severe steric clashes, suggesting that the two folds cannot occur simultaneously in a single TDP-43 molecule. The authors further suggested a disease specific foldome of TDP-43 fibrils, as observed with Tauopathies.

Taken together, these studies suggest that the C-terminal domain of TDP-43 transiently adopts a range of well-ordered shapes, many of which are capable of self-association. Mutations and post-translational modifications may change the kinetics of these states, influencing the balance between fibril formation and dissolution, as implied by Cao et al. (2019) who showed that mutations within the fibril interface delayed aggregation, supporting the argument that mutations will affect which shape is adopted by the CTD.

##### Liquid-liquid phase separation and aggregation

Conflicting data exists for the contribution of the CTD to TDP-43 aggregation. Multiple studies point to the C-terminus essential to aggregation and pathology (Dewey et al., 2010; Guo et al., 2011; Budini et al., 2012; Jiang et al., 2013, 2016; Mompeán et al., 2014; Prasad et al., 2018). Others have shown that the CTD is not sufficient, but rather that the CTD combined with the RRM2 is required for significant accumulation of aggregates in cellular models (Johnson et al., 2008; Yang et al., 2010; Fallini et al., 2012; Wang et al., 2013). And indeed, as discussed above, the RRM domains contain amylogenic sequence elements that contribute to aggregation (Shodai et al., 2012, 2013; Chiang et al., 2016; Guenther et al., 2018b) and constructs lacking the CTD have been shown to aggregate as well (Zacco et al., 2019).

Further complicating the picture is the recent identification of the role of reversible self-association, generally termed LLPS (liquid-liquid phase separation) which is thought to initiate formation of SGs and to which both the NTD and CTD seem to contribute (Conicella et al., 2016; Schmidt and Rohatgi, 2016; Afroz et al., 2017; Li et al., 2018; Wang A. et al., 2018; Wang L. et al., 2018; Babinchak et al., 2019). The conditions under which TDP-43 undergoes LLPS are highly sensitive to the conditions of the experiment (Conicella et al., 2016; Li et al., 2018; Wang L. et al., 2018). Moreover, interaction between the CTD and charged nucleic acids, specifically ssDNA, can increase the CTD’s ability to undergo LLPS, thought to occur *via* the many aromatic and pi interactions (Wang L. et al., 2018). Despite a lack of direct modulation of LLPS formation by charged residues, it was found that arginine residues played an integral role in changing the material properties and dynamics of the droplets formed by TDP-43. Interestingly, Mompeán et al. (2016b) suggested that electrostatic repulsion modulates the formation of TDP-43 aggregation.

The link between LLPS and aggregation is unclear, but several studies provide compelling evidence that time is a key factor. LLPS is important for formation of membraneless organelles, including stress granules [SGs, recently reviewed in Gomes and Shorter (2019)]. As SGs age, they tend to lose their dynamic nature, as a result of formation of protein fibrils thought to contribute to development of irreversible structures (Holehouse and Pappu, 2018; Vogler et al., 2018; Wang A. et al., 2018; Babinchak et al., 2019; Zhang et al., 2019).

In contrast, a recent study showed the ability of TDP-43 to undergo long-lasting liquid-demixing in a stress granule-independent fashion by either increasing cytoplasmic TDP-43, exposure to amyloid-like TDP-43, or arsenite stress (Gasset-Rosa et al., 2019). The authors further show the conversion, upon additional arsenite stress, of those TDP-43 droplets into solid-like structures, that further recruit components of the nucleocytoplasmic transport machinery, leading to impaired nuclear transport. This is thought to accelerate TDP-43 nuclear depletion and result in cell death.

In addition, some mutations within the CTD were shown to alter propensity for LLPS (Jiang et al., 2016), in addition to altering splicing function (Fratta et al., 2018).

### Post-translational Modifications of TDP-43

TDP-43 undergoes a significant number of post-translational modifications (Figure 1). Most of them are associated with pathological TDP-43 and are a hallmark feature of TDP-43 proteinopathy. Another recent review that focuses on a mechanistic perspective has described several post-translational modification of TDP-43 in a context of health and disease (Buratti, 2018). Our goal is to bring a structural point of view on those modifications.

#### Oxidation

**FIGURE 6:**
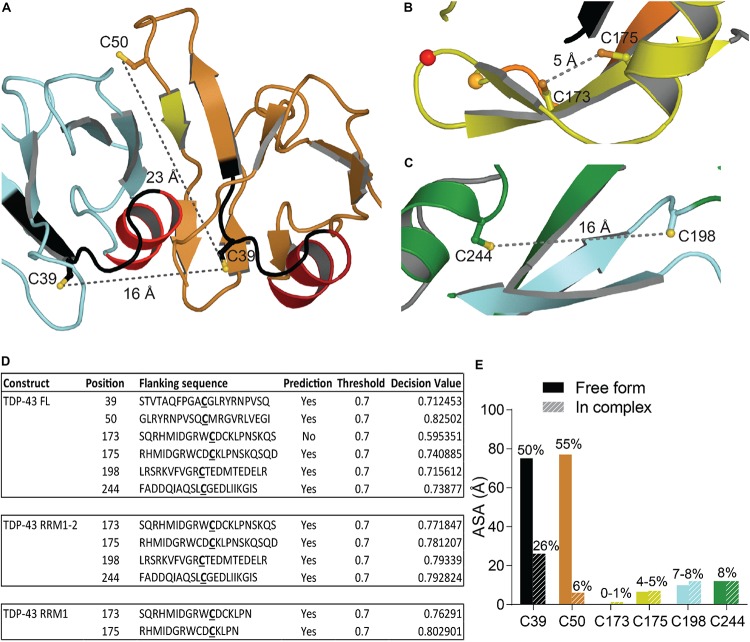
Oxidation of Cysteine residues in TDP-43. **(A)** Distance and localization of C39 and C50 in TDP-43 NTD (PDB code: 5mrg). **(B)** Distance between Cys173 and Cys175 and between Cys198 and Cys244 **(C)** in RRM1-2 (PDB code: 4bs2). **(D)** Table showing accessibility of cysteine residues (Cys39 and Cys50 were calculated using 5mrg, Cys173, 175, 198, and 244 were calculated using 4bs2). The prediction, using the online tool RSCP, validated a residue as likely to be oxidized when the decision value was above the threshold (0.7). **(E)** Accessibility of the cysteine residues calculated with Areaimol as implemented in the CCP4 suite (Otwinowski and Minor, 1997).

Oxidative stress is a hallmark feature of neurodegenerative diseases and ALS was suggested as a cysteninopathia, an aberration of cysteine residues modifications (Valle and Carrì, 2017). It has been hypothesized that the generation of ROS could trigger some TDP-43 pathology (Cohen et al., 2012). The authors treated cells with various stressors that generate ROS using different pathways, i.e., H_2_O_2_, arsenite, heat shock and cadmium, and found a dramatic shift in TDP-43 solubility, attributed to a direct modification of TDP-43 *via* cysteine oxidation. All TDP-43 cysteine residues were suggested as targets: Cys173, Cys175, Cys198, and Cys244 as the major redox−regulated cysteine residues and Cys39 and Cys50 to a much lesser extent. The authors further reported intramolecular and intermolecular disulfide bonding implicating Cys173, Cys175, Cys198, and Cys244. Since Cys39 and Cys50 are about 22–24 Å apart (Figure 6A), intramolecular disulfide bonding between those two residues is very challenging and a study on NTD oxidation did not describe any aggregation (Chang et al., 2013). Cys39 and Cys50 are known to be in the dimeric interface of TDP-43 NTD and mutations of those residues into Serine induced a weaker oligomerization compared to wild-type, suggesting those cysteines to have contribution into assembly (Wang A. et al., 2018).

The distance between Cys173 and Cys175 is 5.1 Å and between Cys198 and Cys244 is 15.5 Å, intramolecular disulfide bond formation seems unlikely on a native TDP-43 structure (Figures 6B,C). An independent study demonstrated the sequential oxidation of RRM1, with Cys173 being preferentially oxidized and leading to a conformational change allowing Cys175 to be modified and a subsequent formation of cross-linked dimers (Chang et al., 2013). It is worth noting that Cys173 is the least accessible of the six cysteine residues and the least likely to be oxidized in the full-length protein, based on predictions by RSC oxidation prediction, an online web server able to predict the occurrence of redox-sensitive cysteine within the protein sequence (Sun et al., 2016; Figure 6D).

Analysis of the tandem RRM1-RRM2 structure (Lukavsky et al., 2013) shows that Cys173 and Cys175 make contacts with residues in the RRM1. Loss of those contacts by oxidation could explain the exposure of amyloidogenic residues 166–173, since Cys173 and 175 were shown to control both correct and aberrant folding of TDP-43 in ALS depending on the freedom of their thiol group (Shodai et al., 2013).

Given the proximity between oxidation sites and the disease-exposed regions of TDP-43 retrieved in ALS patients (166–173 and 246–255) as well as cleavage sites, the early misfolding of TDP-43 by oxidation, upstream of ubiquitination, phosphorylation, and fragmentation, as suggested in Cohen et al. (2012), is an attractive hypothesis. Moreover, disease-linked mutations G348C and S379C, which introduce new cysteine residue in TDP-43, were shown to undergo oxidation, generating cross-linked TDP-43 species, further supporting the role of early oxidation in TDP-43 misfunction (Cohen et al., 2012).

#### Acetylation

Lysine acetylation is a post-translational modification associated with various pathways including RNA processing, cytoskeleton association, and cellular signaling among others [see Narita et al. (2019) for an exhaustive list]. Recently, acetylation has been associated with aggregating proteins such as Tau (Irwin et al., 2012), Huntingtin (Arbez et al., 2017), and SOD1 (Abdolvahabi et al., 2015).

**FIGURE 7:**
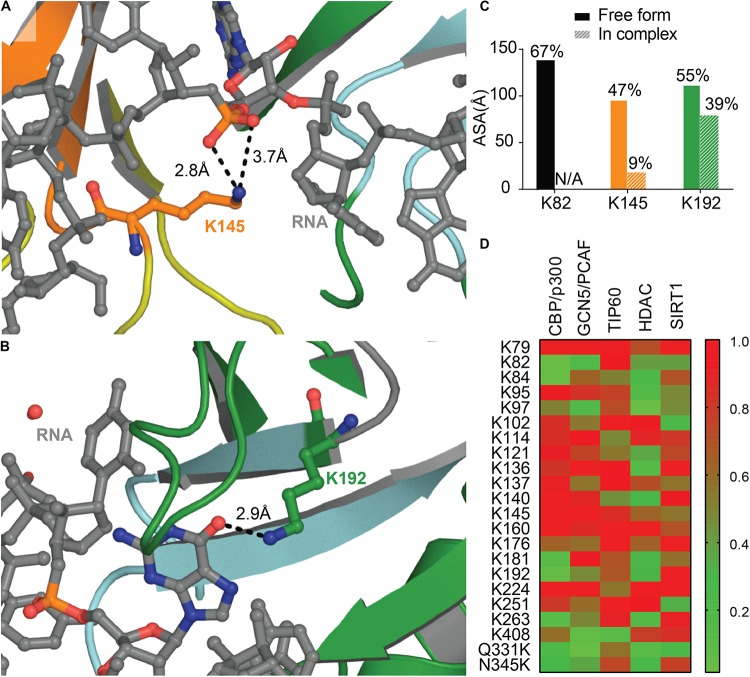
Acetylation of TDP-43 Lysines. Lys 145 in RRM1 (*orange*) **(A)** and Lys 192 in RRM2 (*green*) **(B)** shown in Cohen et al. (2015) to be acetylated are located at the interface with nucleic acid (RNA, *gray*) PDB ID 4bs2 (Lukavsky et al., 2013). **(C)** Accessibility of the lysine residues shown to be acetylated calculated using Areaimol as implemented in the CCP4 suite (Otwinowski and Minor, 1997). **(D)** Prediction of acetylated lysines by different acetylation systems, using ASEB (http://bioinfo.bjmu.edu.cn/huac/; Li et al., 2012, 2013; Zhai et al., 2017) shown as a heat map of *p*-values. For each query, a *p*-value is assigned based on its similarity to known sites. The smaller the *p*-value of the residue, the closer it is to known acetylated sites.

A recent study identified two main sites of acetylation in TDP-43, Lys145, and Lys192 (Figures 7A,B; Cohen et al., 2015). Generating acetylation mimics (K145Q and/or K192Q) led to the formation of nuclear speckles, and cytoplasmic aggregates when the TDP-43-nuclear localization signal was impaired (TDP-43 ΔNLS). The acetylation-null mutant (TDP43-2KR) was diffuse, even when TDP-43 NLS was impaired, supporting acetylation of TDP-43 as a pathological modification of the protein. TDP-43 K145Q mutant exhibited decreased nucleic acid binding that translated into decreased splicing function (Cohen et al., 2015). This is not surprising, K145 is part of RRM1 RNP-1 motif and K192 of RRM2 RNP-2, they are both moderately accessible in the free protein (Figure 7C), and acetylation is a PTM known to modulate protein-nucleic acid binding (Arbely et al., 2011; Ren et al., 2016) by neutralizing the electrostatic interface.

Nevertheless, residues K145 and K192 were not found acetylated in a recent study using mass spectrometry on TDP-43 inclusions in ALS brains of two different patients (Kametani et al., 2016). The authors describe instead Lys82 as being modified, hence suggesting heterogeneity between patients. Acetylation at Lys82, found in TDP-43 NLS, could impair proper shuttling to the nucleus and act as a pathological event. Prediction of acetylation sites using ASEB (A Web Server for KAT-specific Acetylation Site Prediction^1^) (Li et al., 2012, 2013; Zhai et al., 2017) shows a high potential of Lys82 as well as Lys192 (Figure 7D), while K145 were less likely to be modified. The implication of those two Lysines in disease remains to be further examined. We also probed for mutations that could enhance acetylation, Q331K and N345K, and found that they both are predicted as possible sites of acetylation. It would be intriguing to look for these acetylation sites in the brain of patients with these acetylation enhancing mutations.

#### Zinc Binding

A recent study described the ability of zinc ions to bind TDP-43 with an affinity in the micromolar range. Binding of Zinc was shown to decrease TDP-43 thermostability and formed Thioflavin-T-positive aggregates, reminiscent of amyloid nuclei (Garnier et al., 2017). Zinc treated SY5Y neuronal-like cells recapitulated several hallmarks of TDP-43 proteinopathy including reduced expression, formation of small nuclear inclusions, and diffuse cytosolic localization. The treatment, however, did not cause formation of CTD fragments, ubiquitination or phosphorylation of TDP-43 (Caragounis et al., 2010). Although an indirect route was not ruled out, especially *via* the generation of ROS through NMDA- or mitochondrial-mediated pathways by Zn^2+^, zinc ions are also known to bind and promote *in vitro* aggregation of Tau (Huang et al., 2014), alpha-synuclein (αSyn) (Valiente-Gabioud et al., 2012) and Amyloid-β Peptide(Aβ) (Alies et al., 2016). Altered zinc homeostasis is also suggested as a risk factor for several neurodegenerative disorders such as ALS or Alzheimer’s disease [see review (Szewczyk, 2013)]. Even though this is still a matter of debate given the relatively poor affinity of zinc for those proteins (in the micromolar range), direct contribution of zinc to TDP-43 aggregation could lead to complexes actively producing ROS similar to Aβ and αSyn (Atrián-Blasco et al., 2018), and further amplifying toxicity.

The predicted binding sites of Zn^2+^ in TDP-43 RRM domains (Garnier et al., 2017) contain Cys residues, modified upon oxidative stress (Cohen et al., 2012), and especially Cys173 and Cys175 in RRM1 (Chang et al., 2013; Figure 6B) that govern aberrant self-assembly at amyloidogenic cores (Shodai et al., 2013). Even though Zn^2+^ binding is well-known to protect cysteine residues from oxidation, it has been suggested that some metal-binding cysteines could undergo redox modifications (Pace and Weerapana, 2014). Complexing those Cys residues by zinc ions might have an effect similar to oxidation and might lead to misfolding of the protein.

#### SUMOylation

SUMOylation is a post-translational modification where a small ubiquitin-like modifier (SUMO) sequence is added to a Lysine residue within a SUMO-interaction motif, CKXE/D, C being a large hydrophobic amino acid. SUMOylation was suggested to be a pathological event in ALS, since superoxide dismustase 1 (SOD1), Fused in Sarcoma (FUS) and TDP-43 were found SUMOylated (Dangoumau et al., 2013; Foran et al., 2013) which resulted in an increase aggregation of those proteins. In addition, TDP-43 aggregated in inclusions was found SUMO positive following overexpression in mouse primary neurons (Seyfried et al., 2010).

**FIGURE 8:**
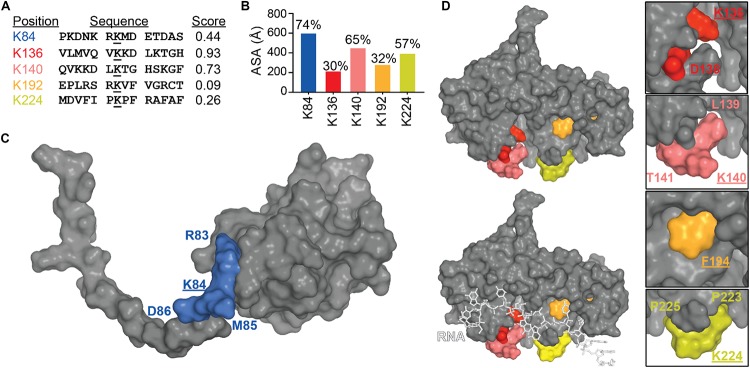
SUMOylation of lysine residues in TDP-43. **(A)** TDP-43 sites that conform to SUMOylation consensus motifs (ranked by Abgent SUMOplot^TM^ Analysis Program). Higher scores indicate greater similarity to positively identified SUMOylation motifs and higher predicted likelihood of endogenous SUMOylation. **(B)** Accessibility of the SUMO motifs at the TDP-43 surface were calculated using Areaimol as implemented in the CCP4 suite (Otwinowski and Minor, 1997) on the NTD structure [PDB ID 5mrg (Mompeán et al., 2017), K84 motif] or the free form of the tethered RRM domains structure [PDB ID 4bs2 (Lukavsky et al., 2013), K136, K140, K192, and K224 motifs]. To note, there is no significant difference in accessibility values when RNA is present. Mapping of the SUMOylation motifs on the surface of TDP-43 NTD **(C)** or RRM1-2 with and without RNA **(D)**.

To date, there is still no confirmation on where the modification occurs on TDP-43, all the SUMOylated sites described in Figure 8A were found in two high-throughput studies (Hendriks et al., 2014; Lumpkin et al., 2017). Prediction site SUMOPlot confirmed K136 as being the most likely to be SUMOylated (Figure 8B) which is consistent with the suggestion in Dangoumau et al. (2013). But given the poor surface accessibility of K136 SUMO motif (30% in the free protein and 27% when TDP-43 is in complex with RNA), SUMOylation at this site is unlikely on a native structure and SUMOylation at residues K84 or K140 seem more likely to occur on a folded TDP-43. While experimental data is necessary to validate the SUMOylation sites, we can speculate that SUMOylation of K84 would disrupt TDP-43 NLS and active transport from cytoplasm to nucleus and SUMOylation at K136 would be the most detrimental by affecting interactions between RRM1 and RRM2 domains as well as RNA binding (Figures 8C,D).

#### Phosphorylation

Abnormal phosphorylation of TDP-43 in ALS has been described as a hallmark feature of cytoplasmic aggregates in ALS/FTLD (Arai et al., 2006; Hasegawa et al., 2008). Indeed, phosphorylation on serine residues 403/404 and/or 409/410 is considered a consistent reference marker of disease (Neumann et al., 2009). Most pathological phosphorylations that occur in the CTD of the protein (Hasegawa et al., 2008; Figure 1), are thought to happen as a later event, and to accumulate on the protein as it is trapped in the cytoplasm over time, depicting attempt from the cellular machinery to trigger degradation (Fang et al., 2014; Kametani et al., 2016; Zhang et al., 2019).

The effect of phosphorylation on TDP-43 remains unclear and largely debated. Several studies point out the propensity of phospho-null mutant to increase aggregation while phospho-mimic mutant had the opposite effect (Brady et al., 2011; Li et al., 2011). On the contrary, another group has reported increased accumulation of TDP-43 following phosphorylation by truncated Casein Kinase 1δ (Nonaka et al., 2016). Phosphorylation at T153 and Y155 by MAPK/ERK Kinase was not associated with protein accumulation and TDP-43 remained soluble but its capacity to bind nucleic acid was reduced, which could be consistent with a second RNA binding site on RRM1 (Li et al., 2017) (see section “RRM-Nucleic Acid Binding”).

Another example is the recently reevaluated ALS mutation, S375G, linked to early onset disease (Newell et al., 2019). This mutation was shown to remove a phosphorylation site resuting in increased nuclear localization compared to wild-type TDP-43. Interestingly, the phosphomimic mutant, S375E, exhibited cytotoxicity and increased cytoplasmic accumulation. The authors suggested the necessity of a reversible phosphorylation in the regulation TDP-43 cellular redistribution.

Finally, about half of the known disease-linked mutations in TDP-43 will either create potential phosphorylated sites (new Ser or Thr residues), remove phoshorylation sites (elimination of Ser or Thr residues) or introduce a phosphomimic residue (Asp and Glu) (Figure 1), arguing that pathogenesis based on phosphorylation alone is possible but is probably correlated with the site of phosphorylation and/or the phosphorylation machinery.

#### Ubiquitination

Ubiquitination was one of the first pathological modifications of TDP-43 that was discovered (Arai et al., 2006; Neumann et al., 2006; Nonaka et al., 2009a). This post-translational modification results in the covalent attachment of a small regulatory protein (8.6 kDa) -ubiquitin- catalyzed by a sequential cascade of enzymes, similarly to SUMOylation, and is well-known for its role into protein degradation.

**FIGURE 9:**
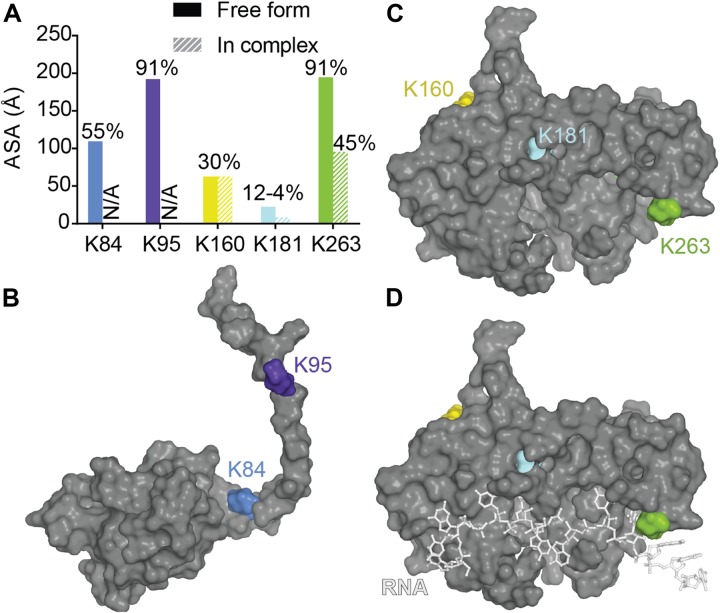
Ubiquitination of lysines in TDP-43. **(A)** Accessibility of the known ubiquitinated Lysine residues were calculated using Areaimol as implemented in the CCP4 suite (Otwinowski and Minor, 1997) on the NTD structure [PDB ID 5mrg (Mompeán et al., 2017), K84 and K95] or the tethered RRM domains structure [PDB ID 4bs2 (Lukavsky et al., 2013), K160, K181, and K263]. Mapping of the SUMOylation motifs on the surface of TDP-43 NTD **(B)** or RRM1-2 with and without RNA **(C,D)**.

A recent mutagenesis study coupled with mass spectrometry was able to find several ubiquitinated sites and suggested a proteasome and autophagosome targeting function of TDP-43 ubiquitination (von Zweydorf et al., 2018). The authors validated K84 and K95 as being modified and showed only K84 to affect nuclear import while K95 seemed to impact CTD phosphorylation. To note, those two modifications seemed to have no effect on TDP-43 solubility. Residues K160, K181, and K263 in the RRM domains, were also validated, but the authors did not pursue further those sites. Mapping of those residues as well as surface accessibility calculations (Figure 9) indicate that (i) K263 is the most accessible to modification (which is consistent with predictions using UbPred^2^) (Radivojac et al., 2010), and modification of this residue could result in direct RNA binding reduction, (ii) K181 modification would be detrimental to TDP-43 structure possibly by modifying interactions between RRM1 and RRM2 especially *via* loss of contact with the critical residue D247 (see RRM domains section for discussion of this residue) or by affecting RRM-NTD interactions since it is close to the linker and (iii) K160 modification would be the less detrimental, even though it has potential contacts with F127 and T157. The authors further noticed a lack of ubiquitination localized in the CTD when considering the full-length TDP-43, which was surprising at first since they previously observed major ubiquitination in a CTF fragment of TDP-43 (residues 193–414) (Hans et al., 2014). Thus, this observation suggests CTD ubiquitination might only occur post TDP-43 cleavage. While ALS-linked mutations Q331K and N345K did not induce further ubiquitination, K263E, very surprisingly led to enhanced ubiquitination that was attributed to ubiquitination to redundant sites (Hans et al., 2014). Interestingly, K263E exhibits higher propensity to form aggregates (Bhandare and Ramaswamy, 2018).

#### Cleavage

An additional consistently observed aberration is the accumulation of fragmented TDP-43 in cytoplasmic aggregates (Neumann et al., 2006; Zhang et al., 2007, 2009; Che et al., 2015; Berning and Walker, 2019). These fragments appear to be the result of caspase cleavage at several sites (Figure 1). Cleavage at D89-A90 results in a 35 kDa fragment (TDP-35) that lacks the NTD and disrupts the NLS but is correctly folded (Che et al., 2015). Sites at D169-G170 and D174-C175 are both associated with the 25 kDa fragment (TDP-25) which lacks the NTD, NLS and most of RRM1 (Li et al., 2015; Chiang et al., 2016; Figure 3B). Additional sites occur at M218-D219 and E246-D247 (Igaz et al., 2009; Nonaka et al., 2009b; Figure 3C). All of these cleavage events lead to disruption or elimination of TDP-43 NLS, trapping the protein in the cytoplasm (Cicardi et al., 2018) and enhancing protein aggregation (Kitamura et al., 2016). Notably, D169-G170, D174-C175, and E246-D247 are located at or in the amyloidogenic cores and as such, cleavage might help in exposing those sequences. Alternatively, early unfolding initiated by other PTMs, for example oxidation of Cys 173 or Cys 244, might result in exposure of those regions, increasing cleavage activity. Further, disease-linked mutations, A90V and D169G (Chiang et al., 2016), link those cleavage sites to pathological forms of TDP-43.

#### PARylation

Poly-ADP-ribose is a post translational modification that conjugates several ADP-ribosyl units by members of the poly(ADP-ribose) polymerase (PARP) family, to generate long and highly negatively charged linear or branched polymers (Teloni and Altmeyer, 2016), in PAR-binding modules. One of the PAR-binding modules is the PAR-binding motif (PBM, typically [HKR]1-X2-X3-[AIQVY]4-[KR]5-[KR]6-[AILV]7-[FILPV]8) where Lys, Asp and Glu can be modified [see (Teloni and Altmeyer, 2016) for full review, and (Gibson and Kraus, 2012) for structural insight]. Multiple PBMs can be found in the same protein and can increase interaction of target protein with PAR conjugation system (Krietsch et al., 2013). PARylation has been linked to DNA damage repair, unfolded protein response and cellular stress response among other [see (Gupte et al., 2017) for a full list and description]. Interestingly, mammalian stress granules have been shown to contain PAR (Catara et al., 2017).

In a search for genetic modifier of TDP-43 toxicity, McGurk et al. (2018) found that downregulation of tankyrase (a PAR catalase) reduced degeneration of the *Drosophila* eye linked to expression of human TDP-43. Sequence alignment led McGurk et al. (2018) to find two regions of interest with 80 and 63% of fitting to the canonical PBM, which turned out to overlap with the bipartite NLS of TDP-43 (Figure 1) and which they experimentally validated. Not only did expression of TDP-43-ΔPBM induce a cytoplasmic localization of the protein, it also excluded the protein from stress granule assembly. Such a PARylation, close to TDP-43 NLS, may serve as steric hindrance to mask TDP-43 NLS and is coherent with the need to avoid active shuttling to nucleus when the protein is needed in stress granules.

Moreover, TDP-43 was able to form LLPS in the presence of PAR while TDP-43-ΔPBM produced an aberrant phase transition. ALS associated TDP-43 fragments (TDP-35 and TDP-25), lacking partially or totally the PAR binding, were not able to co-localize to stress granules nor undergo correct liquid phase demixing.

### Mitochondrial Targeting

**FIGURE 10:**
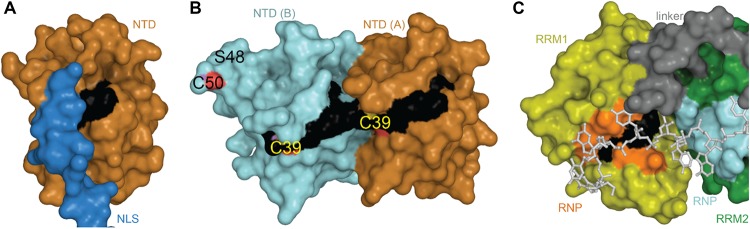
Mitochondrial targeting sequences in TDP-43. **(A)** M1 in the NTD monomeric structure [PDB ID 5mrg (Mompeán et al., 2017)]. **(B)** M1 in the dimeric structure [PDB ID 5mdi (Afroz et al., 2017)] **(C)** M3 in RRM1, overlapping with RNP-1 (*orange*) [PDB ID 4bs2 (Lukavsky et al., 2013)]. Location of mitochondrial targeting signals shown in *black*. RNA is in *light gray*.

Several studies have found TDP-43 localization to mitochondria (Mori et al., 2008; Wang et al., 2016, 2017; Davis et al., 2018) but others have failed to find such an association (Onesto et al., 2016; Kawamata et al., 2017). Nevertheless, one study was able to predict several mitochondrial targeting sequences in TDP-43, named M1-M6 (Wang et al., 2016; Table 2, Figures 1, 10) and experimentally validated three of them as main mitochondrial signals (M1, M3, and M5). Deletion of each of those three signals greatly reduced TDP-43 mitochondrial localization, but did not abolish it, suggesting the need for multiple sequences in the import process. It was further proposed that mitochondrial import of TDP-43 occurred through TOM70/TIM22 complexes, known to mediate translocation of proteins that do not carry a classical matrix-targeting signal and has been shown to bind multiple internal mitochondrial motifs (Backes et al., 2018).

**TABLE 2:** Accessibility of mitochondrial signals.

	**Validated**	**Free protein**	**In complex with RNA**
**M1** 35-FPGACGL-41	Yes	26%	N/A
**M3** 146-GFGFV-150	Yes	13%	1.4%
**M5** 293-RGGGAGLG-300	Yes	N/A	N/A

*Underlined is mutation linked to increased mitochondrial localization.*

With the likely exception of M5, located in the unstructured CTD of TDP-43, none of the mitochondrial signal are surface accessible in the well-folded protein (Table 2). While this is consistent with a very limited -if any- mitochondrial localization of TDP-43 in normal conditions (Wang et al., 2016), the question remains as to how TDP-43 gets translocated in mitochondria in a pathological context. We suggest that several events, among others, could contribute to this state. (1) TDP-43 abnormally interacts with the mitochondrial chaperone Hsp60 in ALS (Freibaum et al., 2010). Fused in sarcoma (FUS) protein, another ALS-linked protein, was also shown to have pathological interaction with Hsp60 which increases its mitochondrial localization (Deng et al., 2015). Increased Hsp60 binding could explain exacerbated mitochondrial localization. (2) The heat shock protein Hsp70 is known to be implicated in mitochondrial import (Young et al., 2003) and to bind TDP-43 as well as dissolve TDP-43 aggregates and rescue toxicity (Udan-Johns et al., 2014; Kitamura et al., 2018). It has been shown that Hsp70 is reduced and mislocalized to aggregates in SOD1 mutants mice (ALS model) (Liu et al., 2005; Chen et al., 2016). Moreover, defects in cytosolic Hsp70 led to enhanced entry of misfolded proteins into mitochondria and elevated mitochondrial stress in yeast, which is believed to translate into humans (Ruan et al., 2017). An abnormal Hsp70 could lead to exacerbated mislocalization of TDP-43 in mitochondria. (3) Interestingly, the M3 mitochondrial sequences is localized at the nucleic acid binding site (RNP-2 of RRM1) and accessibility of the mitochondrial signals are greatly affected by RNA binding (Table 2). Since RNA was shown recently to be neuroprotective (Coyne et al., 2014; Mann et al., 2019), loss of RNA binding could hence promote TDP-43 to localize in mitochondria. (4) Mutations in TDP-43 could contribute to mislocalization to the mitochondria. G298S, occurring in M5, was shown to increase mitochondrial import in patient fibroblasts and cell lines (Wang et al., 2016). This mutation might thus make the mitochondrial signal more accessible. Alternatively, misfolding or unfolding, either local or total, of TDP-43 could help in exposing the mitochondrial targeting sequences and subsequent interaction with mitochondrial import machinery. For example, C39 occurs in M1 and has been shown to be oxidized, which might help in TDP-43 unfolding. Although the crystal structure of the dimeric TDP-43 NTD, which contain dimethylarsino-modified C39 and C50, does show a small increase in the distance between the monomers compared to the dimeric NMR structure, M1 is not significantly more surface exposed (Afroz et al., 2017; Wang A. et al., 2018; Figure 10). Another example would be acetylation at K145, localized just at the N-terminus of the M3 signal and is the first residues of RNP-1 in RRM1. Finally, phosphorylation of TDP-43 was shown to increase mitochondrial localization (Wang et al., 2016) since the phospho-mimic G298D induced increased mitochondrial TDP-43 and phospho-null G298A reduced it.

## Concluding Remarks

TDP-43 is an essential protein, found in all higher eukaryotes (Wang et al., 2004). It is ubiquitously expressed throughout CNS development and into adulthood (Huang et al., 2010), mainly in the nucleus (Wang et al., 2002; Casafont et al., 2009) and in the cytoplasm and mitochondria to a lesser extent. TDP-43 is mislocalized in the cytoplasm of cells in the presence of mutations in the context of neurodegenerative diseases. Possible mechanisms of pathophysiology include mislocalization due to excessive PTMs, aggregation, segregation of RNA in stress granules. All these mechanisms are driven or modulated by effects on the three-dimensional structure of TDP-43.

Solving the three-dimensional structure of the entire protein has been challenging due to large disordered domain and flexible linkers between domains. Differentially dissected constructs of TDP-43 have been used to try to classify TDP-43 interdomain interactions, if any, in order to understand the full protein structure-function relationship. Characterization of these constructs using NMR concluded that TDP-43 has dynamic interdomain interactions and implied an interaction between N-terminus and C-terminus that contributes to its pathology (Wang et al., 2012; Wei et al., 2016). But overall, inter-domain characterization is still underdeveloped. Further structural advances may be possible with Cryo-EM, but the flexible domains will still be problematic. It is more likely that defining direct binding partners of TDP-43 will be more fruitful in defining structural elements not defined in the apo protein. It is also clear from this review, many PTMs will undoubtedly change the protein structure as well as influence binding to other partners.

An additional unanswered question is how full-length protein as higher order oligomers is implicated in RNA splicing. It is typical of RNA-binding proteins with multiple RRM motifs for increased specificity and affinity to form higher order oligomers; hnRNP proteins, for example, bind in tandem to RNA. For TDP-43, polymerization through the NTD has been shown to be implicated in splicing (Afroz et al., 2017). Moreover, while it was shown that TDP-43 CTD is implicated in its splicing function *via* flexible prion-like folding (Wang et al., 2012), a recent study suggested that the ability of TDP-43 to phase separate does not impact its splicing function (Schmidt et al., 2019). A better understanding of the oligomerization foldome of TDP-43 and how it relates to splicing will be necessary to define how the core TDP-43 function (RNA binding) may be modulated by other structural regions.

Another level of complexity arises from the contribution of the CTD to the diseased state that includes a wide variety of mutations clustered in this domain. Indeed, each mutation could contribute to proteinopathy through different mechanisms; no common mechanism has been elucidated thus far. Potentially, mutations in this region could modify the intrinsic properties of the CTD resulting in a shift between LLPS, SG, and aggregate formation of the protein. Alternatively, other mutations could modify the post-translational modification pattern.

From the intense literature on PTM, it is clear that defining the hierarchy between PTMs and various structural motifs from within and from exogenous proteins and how this will influence TDP-43 will be of therapeutic interest. With the advent of better proteomics of patient-derived cells, asking if TDP-43′s extensive PTMs (or which ones) detected in patients correlate with disease onset or if they result from the accumulation of insults will be critical. Coupled with better definition of the structure of TDP-43 and determining if PTM modifications are the cause or a result of the disease will define how we tackle our targeting strategies toward successful therapeutics.

## Author Contributions

All authors have made significant contribution and revised the manuscript critically for important intellectual content.

## Conflict of Interest

The authors declare that the research was conducted in the absence of any commercial or financial relationships that could be construed as a potential conflict of interest.

## Footnotes

1 http://bioinfo.bjmu.edu.cn/huac/

2 http://www.ubpred.org
