# Corrigendum: Structural Insights Into TDP-43 and Effects of Post-translational Modifications

**DOI:** 10.3389/fnmol.2020.00045

**Published:** 2020-04-02

**Authors:** Liberty François-Moutal, Samantha Perez-Miller, David D. Scott, Victor G. Miranda, Niloufar Mollasalehi, May Khanna

**Affiliations:** ^1^Department of Pharmacology, College of Medicine, University of Arizona, Tucson, AZ, United States; ^2^Center for Innovation in Brain Science, Tucson, AZ, United States; ^3^Department of Chemistry and Biochemistry, University of Arizona, Tucson, AZ, United States

**Keywords:** TDP-43 = TAR DNA–binding protein 43, structure, post-translational modification, subdomains, RRM domain

In the original article, there was an error. In “Binding of Zinc was shown to increase TDP-43 thermostability and formed Thioflavin-T-positive aggregates, reminiscent of amyloid nuclei (Garnier et al., 2017).” The word “increase” should be replaced by “decrease”.

A correction has been made to the **Introduction** section, subsection **Post-translational Modifications of TDP-43**, sub-subsection **Zinc Binding**, paragraph **1:**

“A recent study described the ability of zinc ions to bind TDP-43 with an affinity in the micromolar range. Binding of Zinc was shown to decrease TDP-43 thermostability and formed Thioflavin-T-positive aggregates, reminiscent of amyloid nuclei (Garnier et al., 2017). Zinc treated SY5Y neuronal-like cells recapitulated several hallmarks of TDP-43 proteinopathy including reduced expression, formation of small nuclear inclusions, and diffuse cytosolic localization. The treatment, however, did not cause formation of CTD fragments, ubiquitination or phosphorylation of TDP-43 (Caragounis et al., 2010). Although an indirect route was not ruled out, especially *via* the generation of ROS through NMDA- or mitochondrial-mediated pathways by Zn^2+^, zinc ions are also known to bind and promote *in vitro* aggregation of Tau (Huang et al., 2014), alpha-synuclein (αSyn) (Valiente-Gabioud et al., 2012) and Amyloid-β Peptide(Aβ) (Alies et al., 2016). Altered zinc homeostasis is also suggested as a risk factor for several neurodegenerative disorders such as ALS or Alzheimer's disease [see review (Szewczyk, 2013)]. Even though this is still a matter of debate given the relatively poor affinity of zinc for those proteins (in the micromolar range), direct contribution of zinc to TDP-43 aggregation could lead to complexes actively producing ROS similar to Aβ and αSyn (Atrián-Blasco et al., 2018), and further amplifying toxicity.”

The authors apologize for this error and state that this does not change the scientific conclusions of the article in any way. The original article has been updated.
